# Supplementary material for: NMR structure of a vestigial nuclease provides insight into the evolution of functional transitions in viral dsDNA packaging motors
Source: Nucleic Acids Res. 2020 Oct 21;48(20):11737–49. doi: 10.1093/nar/gkaa874 (PMC7672431; doi:10.1093/nar/gkaa874)
Supplement: gkaa874_Supplemental_File [file gkaa874_supplemental_file.pdf]

Supplementary Materials

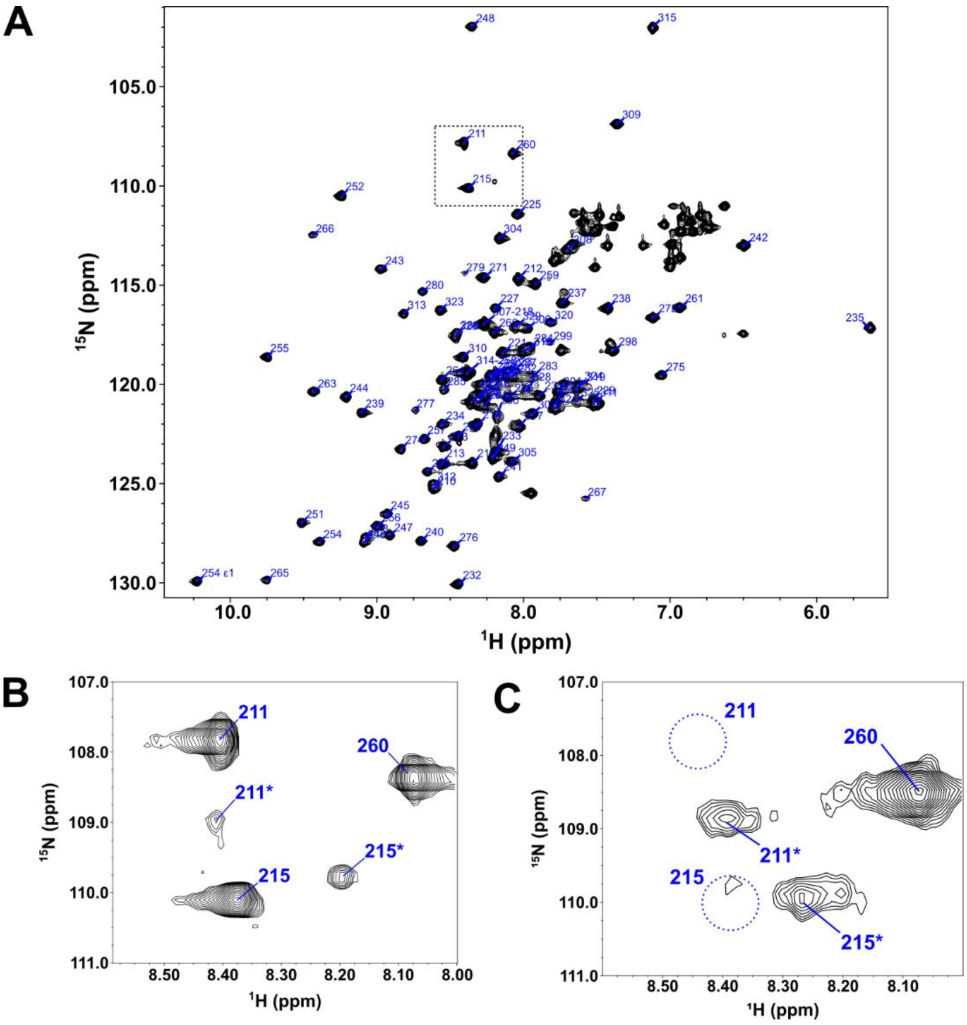

Figure S1. NMR Spectrum of phi29 gp16 (208-332). (A) The ^{1}H-^{15}N HSQC of the phi29 gp16 (208-332) with backbone peaks labeled by residue number. The tryptophan side chain epsilon proton-nitrogen pair is also labeled. **(B)** Inset of the HSQC from the boxed region in (A). This region illustrates the extra peaks from the N-terminal tail described in the text. **(C)** An HSQC collected on an older gp16 sample. The region of the HSQC is the same shown in (B).

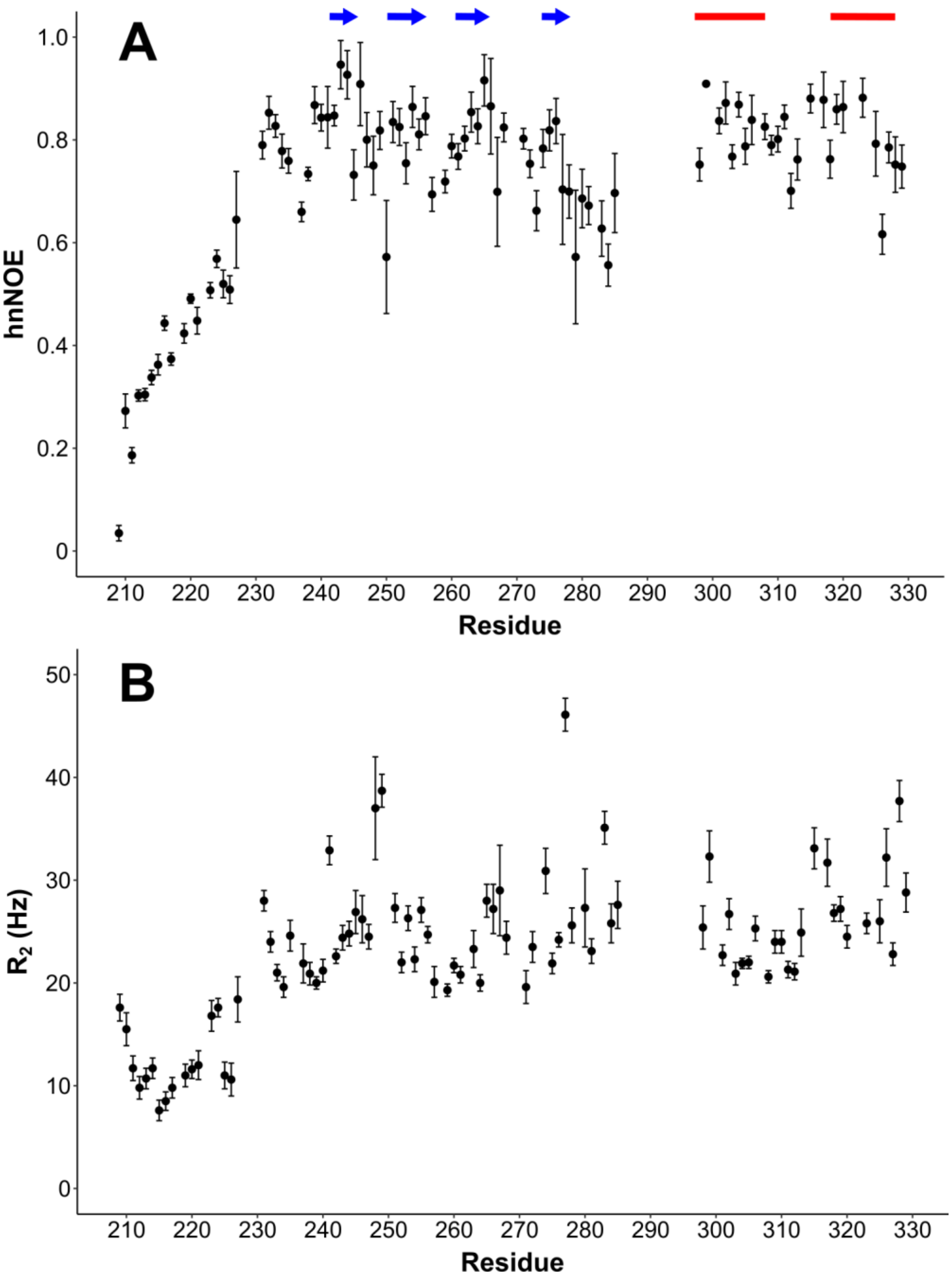

Figure S2. Backbone Dynamics of phi29 gp16 (208-332). (A) Heteronuclear NOE (hnNOE) of the CTD-L. This data is sensitive to fast time scale motions (ps-ns) with rigidity increasing with increasing hnNOE values. A hnNOE value < 0.65 is considered to be flexible. **(B)** Relaxation data from the CTD-L. Higher than average spin-spin relaxation rates (R_2) are indicative substantial signal broadening caused by exchanging structural states on NMR timescale (μs-ms). Residues in secondary structure elements are indicated by blue arrows (β-sheets) and red bars (α-helix) at the top of the figure.

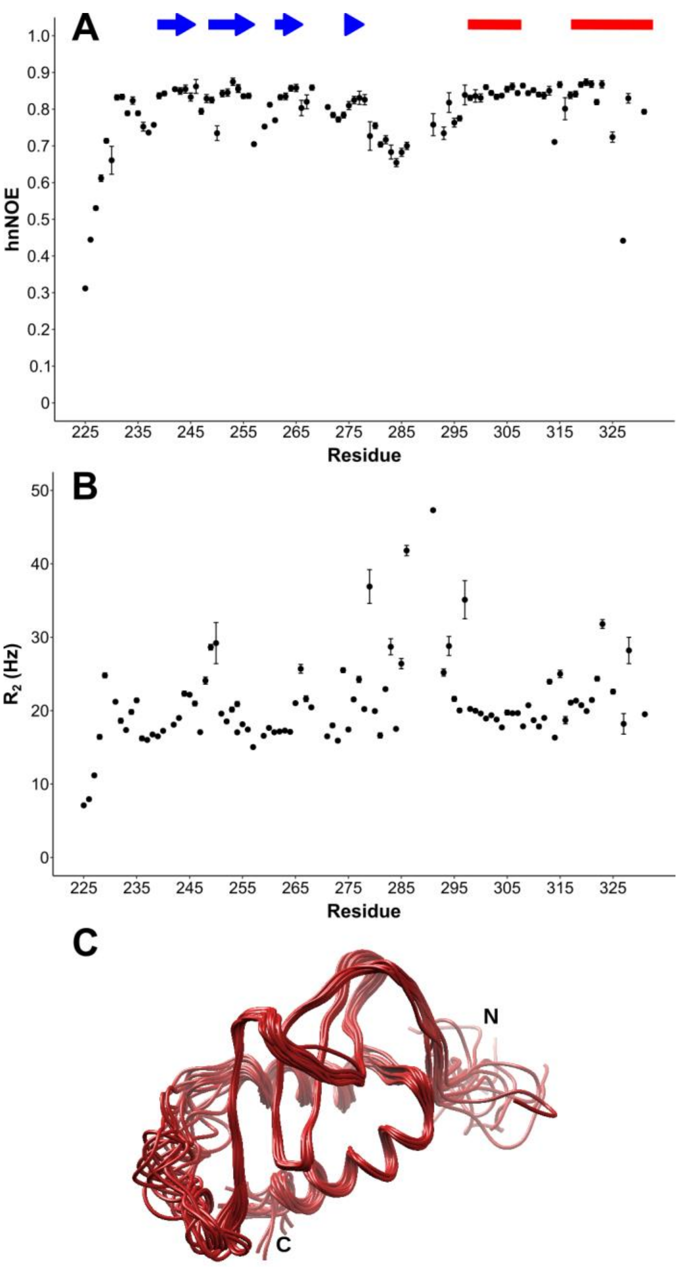

Figure S3. Backbone Dynamics of phi29 gp16 (223-332). (A) Heteronuclear NOE (hnNOE) of the CTD. **(B)** Relaxation data from the CTD. The data collectively highlight the flexibility of the N- and C-terminal tails, as well as the dynamics of the flexible loop between the 4th β-strand and the 1st α-helix (residues 282-296). **(C)** The 15-structure ensemble of the CTD.

Table S1 Structure Statistics for phi29 gp16 (223-332)

**Experimental Restraints**	
Long Range NOE (5 < i - j)[1]	238
Medium Range NOE (1 < i - j ≤ 5)[1]	111
Sequential NOE	171
Backbone Dihedral Angle	212
Hydrogen Bond	44
Total Restraints	776
**Ensemble Statistics (15 structures)**	
RMSD Distance and Ideal Geometry Restraints	
Distance (Å)	0.061±0.002
Bond Length (Å)	0.016
Bond Angle (°)	0.967±0.017
Bond Improper (°)	0.508±0.029
Ramachandran Statistics (MolProbity)	
Favored Region (%)	91.4
Allowed Region (%)	98.5
Disallowed Region (%)	1.5
RMSD from Average Structure	
Backbone Atoms (Secondary Structure)[2]	0.58
All Heavy Atoms (Secondary Structure)[2]	1.1

[1]i-j is the distance of two residues in sequence space

[2]Secondary structures residues are defined as 242-245,250-256,261-265,274-278,297-308,318-328

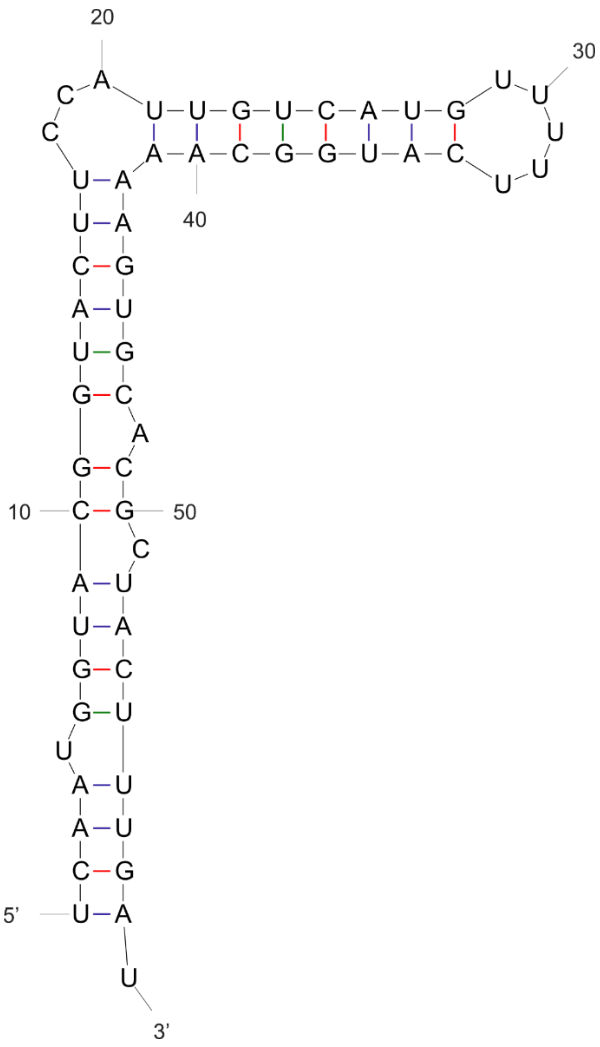

Figure S4. **A secondary structure diagram of the pRNA mimic**. The secondary structure was predicted using the mFold web server (1).

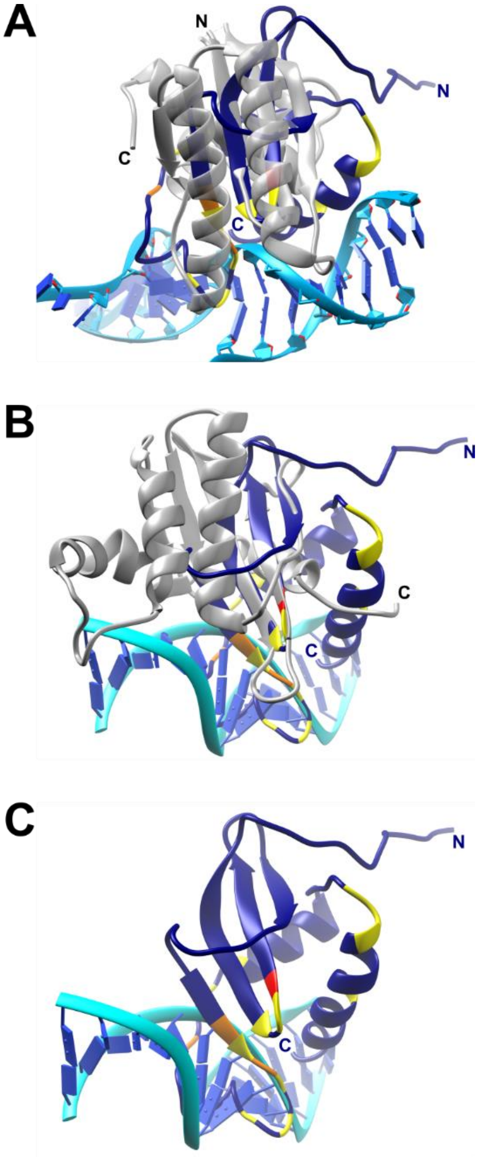

Figure S5. Superimpostions of CTD with RNase H family bound to nucleic acid substrate. (A) The superimposition of CTD with *T. thermophiles* RuvC bound to a Holiday Junction (2)(PDB: 4LD0). The front helices of the RuvC are partially transparent to better visualize the beta-sheet. **(B)** The superimposition of CTD to RNase H1 bound to an RNA/DNA hybrid (3)(PDB: 2QKK). **(C)** The same superimposition as (B) with the RNase H1 removed. The residues are colored by their CSP with dsDNA bound (Fig. 5C): >1 std. dev. (yellow), >2 std. dev. (orange), >3 std. dev. (red).

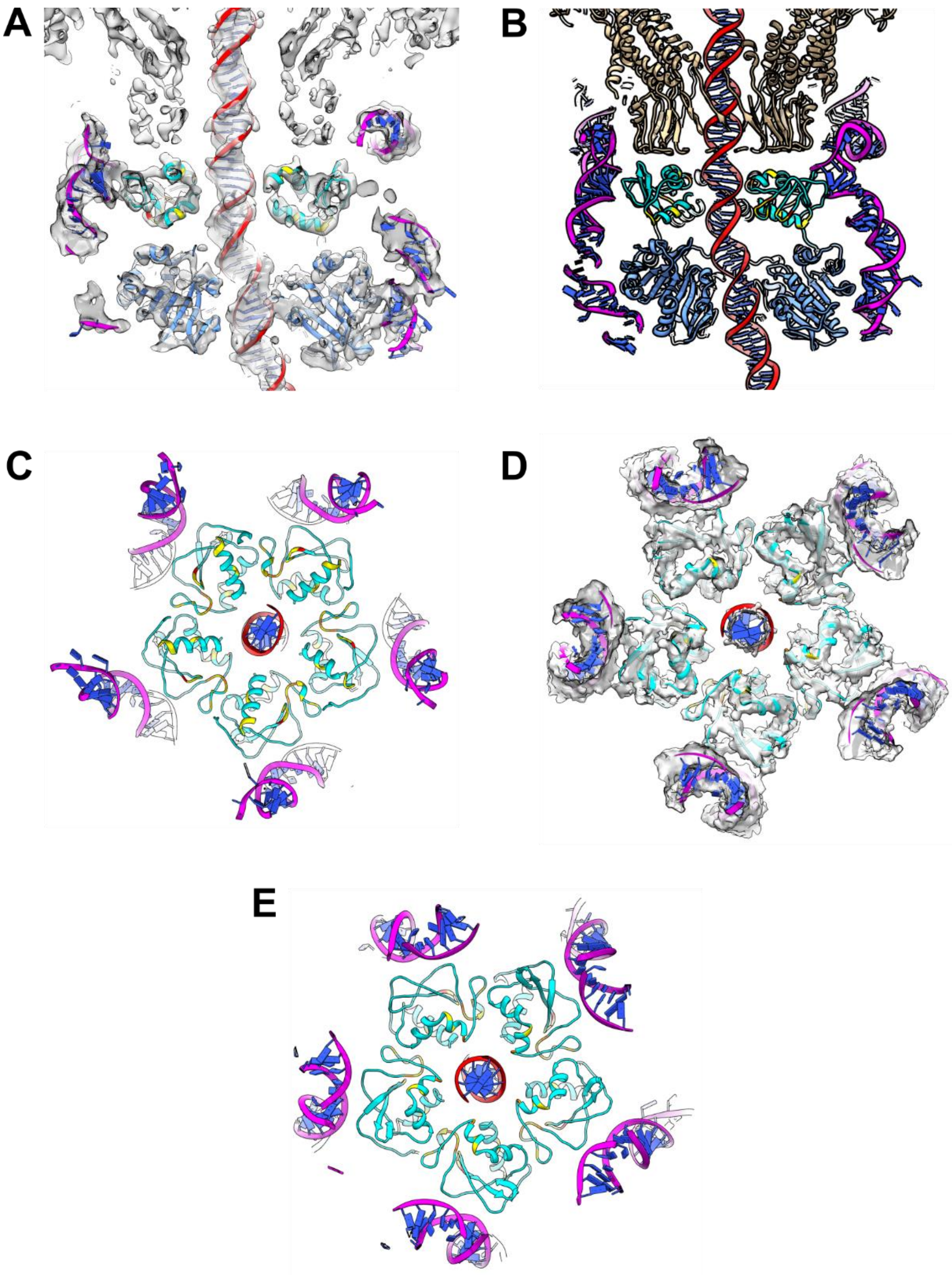

Figure S6. High-resolution structures of phi29 motor components fit to cryoEM density. (A) A side-view of the CTD fitted in its corresponding model. **(B)** The same view as (A) where the density has been removed to facilitate viewing. **(C)** A view from the bottom of the CTD towards the procapsid with the density removed. The view is the same as Fig 5H. **(D)** A view from the top of the CTD towards the NTD. **(E)** The same view as (D) with the density removed. The NMR structure of the CTD is shown in cyan with

DNA-interacting residues identified in CSP data colored as in Fig S5. The crystal structures of the NTD (4)(PDB: 5HD9) is shown in medium blue and the connector protein (5)(PDB: 1FOU) is shown in khaki. The models of the pRNA (6)(PDB: 3R3F) and of B-form DNA are shown in magenta and red respectively, with bases colored blue.

References

1. Zuker,M. (2003) Mfold web server for nucleic acid folding and hybridization prediction. *Nucleic Acids Res.*, **31**, 3406–3415.

2. Górecka,K.M., Komorowska,W. and Nowotny,M. (2013) Crystal structure of RuvC resolvase in complex with Holliday junction substrate. *Nucleic Acids Res.*, **41**, 9945–9955.

3. Nowotny,M., Gaidamakov,S.A., Ghirlando,R., Cerritelli,S.M., Crouch,R.J. and Yang,W. (2007) Structure of Human RNase H1 Complexed with an RNA/DNA Hybrid: Insight into HIV Reverse Transcription. *Mol. Cell*, **28**, 264–276.

4. Mao,H., Saha,M., Reyes-Aldrete,E., Sherman,M., Woodson,M., Atz,R., Grimes,S., Jardine,P. and Morais,M. (2016) Structural and Molecular Basis for Coordination in a Viral DNA Packaging Motor. *Cell Rep.*, **14**, 2017–2029.

5. Simpson,A.A., Tao,Y., Leiman,P.G., Badasso,M.O., He,Y., Jardine,P.J., Olson,N.H., Morais,M.C., Grimes,S., Anderson,D.L., *et al.* (2000) Structure of the bacteriophage φ29 DNA packaging motor. *Nature*, **408**, 745–750.

6. Ding,F., Lu,C., Zhao,W., Rajashankar,K.R., Anderson,D.L., Jardine,P.J., Grimes,S. and Ke,A. (2011) Structure and assembly of the essential RNA ring component of a viral DNA packaging motor. *Proc. Natl. Acad. Sci.*, **108**, 7357–7362.
